# Photopic Adaptation Mimicked by Y_2_O_3_-Based Optoelectronic Memristor for Neuromorphic Visual System

**DOI:** 10.3390/nano15080579

**Published:** 2025-04-11

**Authors:** Jiajuan Shi, Shanshan Qiao, Xuanyu Shan, Zhuangzhuang Li, Zhipeng Li, Chunliang Wang, Ye Tao, Xiaoning Zhao, Ya Lin, Zhongqiang Wang

**Affiliations:** Key Laboratory for UV Light-Emitting Materials and Technology, Northeast Normal University, Ministry of Education, 5268 Renmin Street, Changchun 130024, China; shijj969@nenu.edu.cn (J.S.); ssqiao@nenu.edu.cn (S.Q.); danxy453@nenu.edu.cn (X.S.); lizz834@nenu.edu.cn (Z.L.); lizp394@nenu.edu.cn (Z.L.); taoy506@nenu.edu.cn (Y.T.); zhaoxn430@nenu.edu.cn (X.Z.); wangzq752@nenu.edu.cn (Z.W.)

**Keywords:** visual adaptation, optoelectronic memristor, neuromorphic vision system

## Abstract

Visual adaptation is one of the most significant features that helps organisms process complicated image information in time-varying environments. Emulating this function is highly desirable for energy-efficient image perception. In this work, we demonstrate an yttrium oxide (Y_2_O_3_)-based optoelectronic memristor and emulate photopic adaptation behavior in a single device. Decay amplitude and photosensitivity are indexed to describe the time-dependent characteristics of photopic adaptation. An intensity-dependent characteristic, namely Weber’s law, is also investigated in this work. Photopic adaptation originates from the trapping of photogenerated carriers in oxygen vacancies. Based on photopic adaptation behavior, a neuromorphic vision system capable of adapting to environmental brightness is constructed using the proposed optoelectronic memristor array. Memristor arrays can emulate sensing and adaptation functions in order to enhance images against bright backgrounds. Our work provides a feasible pathway toward self-adaptive neuromorphic vision systems.

## 1. Introduction

Due to the multifunctional integration of image sensing, storage, and processing for next-generation human–machine interactions and humanoid robots [1,2,3,4,5,6,7,8], bio-inspired neuromorphic vision systems have gained considerable attention in recent years. Conventional artificial visual systems are usually composed of different functional units according to their architectural design and manufacturing technologies. A lack of neuromorphic function and the separation of the physical architecture would result in both significant energy consumption and data delay problems [9,10,11]. In contrast, neuromorphic visual systems with an in-sensor computing architecture have considerable potential in highly efficient image perception. The development of a novel neuromorphic visual system is at the cutting edge of research in this field, exhibiting great promise for applications in neuromorphic intelligence technologies and machine vision [11,12,13].

In biological visual systems, the visual adaptation function is an important feature in achieving autonomic responses to environmental stimuli. This ability is beneficial in adapting to varying light environments [14,15,16]. For instance, an individual might experience temporary dizziness due to retinal overstimulation when suddenly exposed to bright light. Then, the retina gradually reduces its sensitivity over time, enabling improved visual perception. The process by which sensitivity gradually decreases under continuous light stimulation is photopic adaptation. Moreover, the perceived change is proportional to the stimuli intensity, which is known as Weber’s law [17,18,19], which is the critical capacity of the organism to identify and distinguish diverse perceptual stimuli from the background. Visual adaptation enables the human eye to perform detailed visual information analysis in a time-varying environment. However, in conventional machine vision systems, the adaptive processing function usually requires complex hardware circuitry and algorithms, which typically limits the operating efficiency [20,21]. Therefore, developing novel neuromorphic hardware to implement these biological adaptabilities is greatly desired for highly efficient visual perception. To date, much effort has been made in constructing optoelectronic devices to demonstrate visual adaptive processing functions [22,23,24,25,26]. He et al. proposed a photo-triggered organic active adaptation transistor (OAAT) and emulated visual active adaptation by combining photovoltaic- and field-effect modulation [23]. The OAAT exhibits excellent stability and uniformity, and this approach can be applied to a wide variety of organic semiconductors. Liao et al. reported a vision sensor array that uses a bottom-gate bilayer MoS_2_ phototransistor; they demonstrated both scotopic and photopic adaptation by introducing trap states, offering a broad perception range and image contrast enhancement [26].

In this work, we reported a two-terminal memristor with photopic adaptation behaviors by utilizing the nanocomposite film of sodium alginate (SA) and Y_2_O_3_ NPs. The time-dependent characteristic of photopic adaptation is investigated by experimentally measuring decay amplitude and photosensitivity (*P_t_*) values. In addition, the intensity-dependent characteristic of visual adaptation, i.e., Weber’s law, is also investigated in this work. The above photopic adaptation can be attributed to the trapping and detrapping behaviors of photogenerated electrons in the Y_2_O_3_. Furthermore, an optoelectronic memristor array with photopic adaptation behavior was fabricated, substantially improving the efficiency and accuracy of the image recognition process. The Y_2_O_3_-based optoelectronic memristor can be regarded as a promising candidate device for constructing self-adaptive neuromorphic vision systems.

## 2. Materials and Methods

Device Fabrication:

Memristors with Au/SA: Y_2_O_3_ NPs /FTO sandwich structures were fabricated on ITO substrates and then patterned into optoelectronic memristive arrays with a diameter of 500 μm using a metal mask. SA powder and Y_2_O_3_ NPs powder was purchased from Macklin Biochemical Co., Ltd., Shanghai, China. First, SA powder (100 mg) and Y_2_O_3_ NPs (200 mg) were mixed in deionized water (10 mL). The solution was mechanically stirred at room temperature for a long time (≥3 h), allowing the powder to dissolve fully and the SA: Y_2_O_3_ NPs nanocomposite film to be formed. The film was fabricated by spin-coating SA: Y_2_O_3_ NPs solutions on the ITO/glass substrate with a spin speed of 3000 rpm and spin time of 30 s. Finally, the Au electrodes were deposited on the top of the film to act as electrodes by using thermal evaporation methods.

Measurement and Characterization:

The optical signals were measured using a xenon lamp (LA-410UV, Hayashi, Tokyo, Japan) with an optical filter. Device conductance was monitored with a source meter (2636b, Keithley, Solon, OH, USA) and a probe station (TTPX, Lake Shore, Westerville, OH, USA). The positive current was defined as the flow from the top Au electrode to the bottom ITO electrode. The Y_2_O_3_ nanoparticles in mechanism part were annealed at 500 °C in a muffle furnace under an air atmosphere for 3 h.

## 3. Results and Discussion

**Figure 1:**
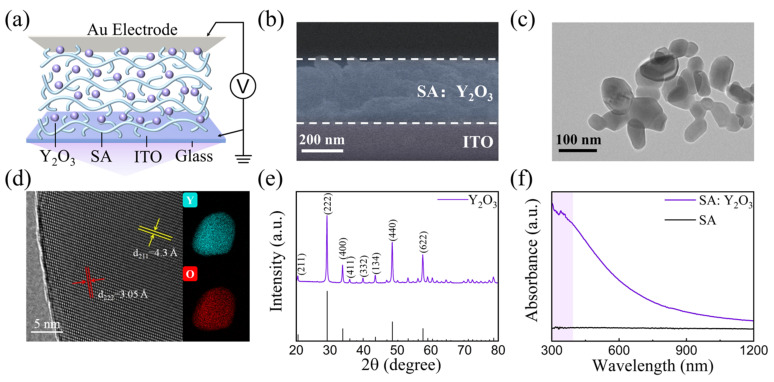
(**a**) Schematic diagrams and (**b**) cross-sectional SEM image of Au/SA: Y_2_O_3_/ITO optoelectronic memristor; (**c**) TEM and (**d**) HRTEM images; (**e**) XRD pattern of Y_2_O_3_ NPs; and (**f**) absorption spectra of SA: Y_2_O_3_ nanocomposite and pure SA films.

As shown in Figure 1a, the optoelectronic memristor consists of an Au/SA: Y_2_O_3_ NPs/indium tin oxide (ITO) sandwich structure. A cross-sectional scanning electron microscopy (SEM) image of SA: Y_2_O_3_ is presented in Figure 1b; the film thickness is about 300 nm. In addition, Au films were deposited using thermal evaporation methods, and these served as the top electrodes (TEs). A read voltage of 0.5 V was applied to the Au top electrode, while the bottom ITO electrode was grounded, and the optical signal was applied to the device through the top electrode. As shown in the transmission electron microscopy (TEM) images (Figure 1c,d), Y_2_O_3_ NPs with a spherical shape exhibit an average particle size of ~50 nm. The lattice fringes of 0.430 and 0.305 nm correspond to the (211) and (222) planes of anatase Y_2_O_3_, respectively. Then, the crystallographic structure of Y_2_O_3_ NPs was uncovered by XRD measurements, as shown in Figure 1e. Y_2_O_3_ NPs have eight broad peaks at 20.5°, 29.1°, 33.8°, 35.9°, 39.8°, 43.5°, 48.5°, and 57.6°, corresponding to the (211), (222), (400), (411), (332), (134), (440), and (622) planes, respectively; this is consistent with the cubic phase of Y_2_O_3_ [27,28,29]. Figure 1f illustrates the absorbance spectra of SA: Y_2_O_3_ NPs nanocomposite and pure SA films. Using the SA film as a reference, the SA: Y_2_O_3_ NPs film exhibits strong light absorption at wavelengths smaller than 390 nm [30,31,32]. Therefore, in this work, optical modulation was performed using a xenon lamp with a UV (360–390 nm) optical filter.

**Figure 2:**
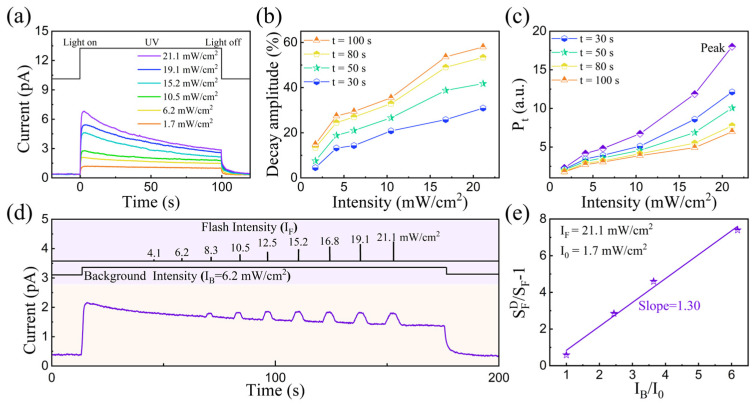
(**a**) Real-time current response of memristor to various light stimuli under fixed voltage of 0.5 V; (**b**) correlation between decay amplitude and light intensity; (**c**) correlation between time-dependent photosensitivity (*P_t_*) and light intensity; (**d**) current response dependent on flash stimulation intensity (from 4.1 to 21.1 mW/cm^2^). Intensity of background light stimulation is fixed at 6.2 mW/cm^2^; (**e**) flash sensitivity *S_F_* of device as function of background intensity *I_B_*.

As a critical feature of biological retina, visual adaptation comprises two primary forms, i.e., scotopic and photopic adaptation [33,34,35]. In this work, photopic adaptation is implemented using the characteristic that the output current decreases over time under high-intensity UV irradiation. Figure 2a shows the photoresponsivity evolution of our device under different irradiation conditions. In this work, the photocurrent was recorded under a fixed voltage of 0.5 V, contributing to its ultra-low power consumption (pW level). The memristor current exhibits a gradual increase at a light intensity of 1.7 mW/cm^2^. By contrast, the output current increases initially and then decreases gradually within the following few seconds, when the optical intensity increases to 6.2 mW/cm^2^. The above process is similar to photopic adaptation in biological visual systems. The devices have excellent uniformity across 50 continuous cycles (Supplementary Material, Figure S1), providing a stable basis for implementing photopic adaptation in future studies. In this case, the time-dependent decay amplitude and photosensitivity (*P_t_*) are defined as follows:

*Decay amplitude* = (*I_peak_* − *I_t_*)/*I_peak_* × 100%

*P_t_* = Δ*I_t_*/*I_dark_*

where *I_peak_* represents the transient output current under light stimulus, *I_t_* is the current after ‘t’ seconds of light irradiation, and *I_dark_* is the dark current. Then, we evaluated the photopic adaptation performance after light exposure for ‘t’ seconds (Supplementary Material, Figure S2) [23,36,37]. It can be seen that the *decay amplitude* increased with an increase in light intensity, as shown in Figure 2b; this is analogous with the photopic adaptation feature. The above result indicates that photopic adaptation is preferentially activated under high-intensity optical stimulus, protecting the receptors from fatigue [37,38]. In addition, as shown in Figure 2c, the photosensitivity after light exposure at 21.1 mW/cm^2^ for 30, 50, 80, and 100 s decreased from 18 to 12.1, 10.1, 7.6, and 7.0, respectively, consistent with the photopic adaptation feature.

Moreover, Weber’s law is emulated by introducing additional flash stimulations with *I_F_* varying from 8.3 to 21.1 mW/cm^2^, as shown in Figure 2d and Supplementary Material Figure S3. The background intensity *I_B_* is desensitized to steady states. It can be seen that the device current is only sensitive to flash stimulation as *I_F_* is larger than *I_B_*. This means that the device sensitivity of flash stimulation strongly depends on the background condition. The proportional relationship between sensitivity changes and background intensity is known as Weber’s law, which is expressed as follows:

(1) $$\frac{S_{F}^{D}}{S_{F}}-1=\frac{I_{B}}{I_{0}}$$

where $S_{F}^{D}$ and *S_F_* are the flash sensitivity in darkness and under background conditions, respectively. *I_0_* is equal to the background intensity required to reduce the sensitivity by half [37,38,39]. According to our experimental results (Supplementary Material, Figure S4), the constants $S_{F}^{D}$ and *I_0_* are calculated as 0.1 and 1.7 mW/cm^2^, respectively. Figure 2e shows the curve of Weber’s law according to Equation (1), where *S_F_* is inversely proportional to *I_B_* with a slope of 1.30, resembling that of biological receptors.

**Figure 3:**
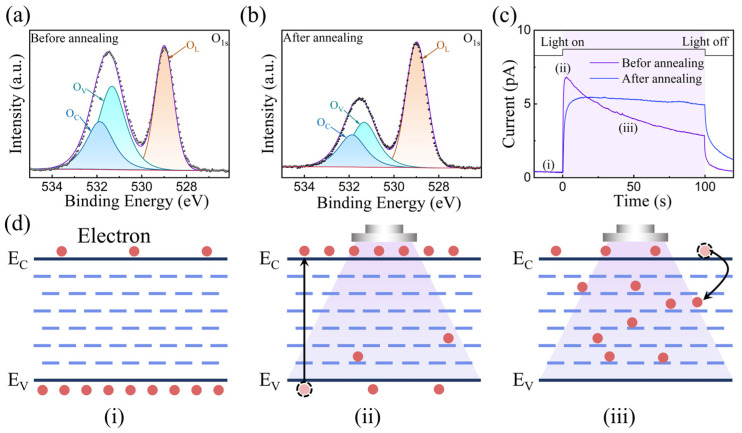
X-ray photoelectron spectroscopy (XPS) of Y_2_O_3_ before (**a**) and after (**b**) annealing. (**c**) Photocurrent response under UV irradiation for Y_2_O_3_-based memristor before/after annealing; (**d**) schematic diagrams of photopic adaptation mechanisms. (**i**) the initial state before receiving the light, (**ii**) the current peak state induced by light, (**iii**) the adaptive process by reducing the current to a certain level.

We also investigated the operating mechanism of our Y_2_O_3_-based optoelectronic memristor. According to Figure 1d and Figure 2a, light-induced adaptation behavior can be attributed to the Y_2_O_3_ NPs in the nanocomposite film. Generally, the characteristics of oxide films are closely associated with their chemical compositions [40]. X-ray photoelectron spectroscopy (XPS) was used to investigate the surface composition and chemical states of Y_2_O_3_ nanoparticles. As shown in Figure 3a,b, the O1s XPS spectra of Y_2_O_3_ nanoparticles before and after annealing are fitted into three components. The peaks located at 529.0, 531.3, and 531.8 eV correspond to lattice oxygen (O_L_), oxygen vacancies (O_V_), and chemisorbed oxygen (O_C_), respectively [41,42,43]. A large amount of O_V_ exists in the Y_2_O_3_ nanoparticles before annealing; however, after annealing, the amount of O_V_ clearly decreases. Figure 3c shows the responsivity evolution of our device before and after annealing. Before annealing, the response current rises abruptly when the device is irradiated by UV light and then decays gradually, indicating photopic adaptation. By contrast, Au/Y_2_O_3_(annealed)/ITO exhibits a gradual increase in response current under the same UV light. These results suggest that the photopic adaptation behavior can be attributed to electron trapping/detrapping in O_V_. As shown in Figure 3d, upon light irradiation, electrons in the valence band and trap states are promptly excited into the conduction band, accompanied by a synchronously enhanced photocurrent. However, some photogenerated carriers will be captured by the trap sites in Y_2_O_3_, causing photocurrent attenuation. Thermal treatment reduces inherent trap sites effectively, thereby reducing the trapping probability of photogenerated electrons. As above, photopic adaptation behavior is absent in annealed devices [44,45,46].

**Figure 4:**
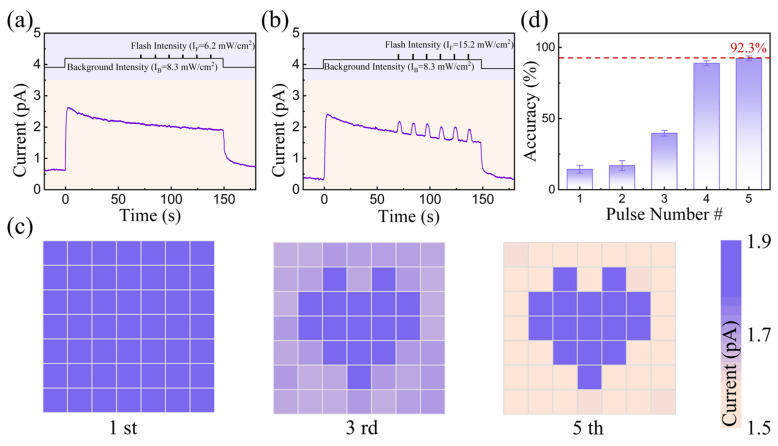
(**a**) The response current with flash intensities of 6.2 mW/cm^2^ and (**b**) 15.2 mW/cm^2^ against a bright background (8.3 mW/cm^2^); (**c**) pattern recognition against a bright background in a 7 × 7 optoelectronic memristive array. With a continuous light background, the ‘heart-shaped’ pattern is gradually identified from the bright background; (**d**) the recognition accuracy as a function of the pulse number. The recognition accuracy of the image can be improved from 14.34% to 92.32%, with the light pulse number increasing to 5.

In addition, in our memristive array, image sensing and adaptation behavior under a bright background can be emulated in our memristive array. We constructed a 7 × 7 device array to perceive the ‘heart-shaped’ pattern under a bright light background (Supplementary Material Figure S5); a light stimulus of 8.3 mW/cm^2^ was applied to all 7 × 7 pixels as a bright background. Light signals with different intensities (6.2 and 15.2 mW/cm^2^, 2 s) were applied to parts of the units as ‘heart-shaped’ patterns, respectively, as illustrated in Figure 4a,b. For a flash intensity of 6.2 mW/cm^2^, the ‘heart-shaped’ patterns could not be identified from the bright background conditions (Supplementary Material Figure S6) because the device is only sensitive to flash stimulations, as *I_F_* is larger than *I_B_* in the photopic adaptation state. For a flash intensity of 15.2 mW/cm^2^, the device array with a larger response current value was saturated in the instantaneous exposure environment, hindering image recognition. Interestingly, the ‘heart-shaped’ patterns could be gradually identified from the bright background conditions due to the decreased sensitivity under continuous optical irradiation, as shown in Figure 4c. The recognition accuracy of images against bright backgrounds can be improved from 14.34% to 92.32%, with the light pulse number increasing to 5, as shown in Figure 4d. These results indicate that our device can be utilized as exposure control elements for the next generation of intelligent circuits, exhibiting considerable potential for application in artificial intelligence, i.e., autonomous driving, and vision.

## 4. Conclusions

In summary, we demonstrated a Y_2_O_3_-based optoelectronic memristor and emulated the photopic adaptation of biological retina. Moreover, the time-dependent nature of photopic adaptation and the changing sensitivity, dependent on background conditions (i.e., Weber’s law), were also investigated. The corresponding mechanism can be attributed to the trapping of photogenerated electrons in oxygen vacancies. We emulated image sensing and adaptation functions based on photopic adaptation. Our work provides a promising hardware platform for future self-adaptive neuromorphic vision systems and human–machine interfaces.

## Data Availability

The data that support the findings of this study are available from the corresponding authors upon reasonable request.

## Supplementary Materials

The following supporting information can be downloaded at: https://www.mdpi.com/article/10.3390/nano15080579/s1, Figure S1: The statistical distribution of the *I_peak_* and *I_steady_* variability for 50 continuous cycles. Figure S2: Definition of time-dependent photosensitivity (*P_t_*) and Decay amplitude, where *I_dark_* is the dark current, It is the current after t seconds of light irradiation and *I_peak_* represents the under light stimulus. Figure S3: Current response of Y_2_O_3_-based optoelectronic memristor to background stimulation (intensity of 1.7 and 4.1 mW/cm^2^) and flash stimulations (intensity from 4.1 to 21.1 mW/cm^2^). Figure S4: (a) Current response of Y_2_O_3_-based optoelectronic memristor under background stimulation of 0 mW/cm^2^. (b) Correlation between sensitivity and background intensity. Figure S5: A photograph of a 7 × 7 optoelectronic memristive array with each pixel having an area of ~0.78 mm^2^. Figure S6: The process of recognizing the ‘heart-shaped’ pattern under a bright background (8.3 mW/cm^2^) with a flash intensity of 6.2 mW/cm^2^ in a 7 × 7 device array. With continuous light background, the ‘heart shape’ pattern cannot be identified from the bright background conditions.

## References

[1] Zhu Q., Li B., Yang D., Liu C., Feng S., Chen M., Sun Y., Tian Y., Su X., Wang X. (2021). A flexible ultrasensitive optoelectronic sensor array for neuromorphic vision systems. Nat. Commun..

[2] Kim M.S., Kim M.S., Lee G.J., Sunwoo S.H., Chang S., Song Y.M., Kim D.H. (2022). Bio-Inspired Artificial Vision and Neuromorphic Image Processing Devices. Adv. Mater. Technol..

[3] Choi C., Lee G.J., Chang S., Song Y.M., Kim D.H. (2024). Inspiration from Visual Ecology for Advancing Multifunctional Robotic Vision Systems: Bio-inspired Electronic Eyes and Neuromorphic Image Sensors. Adv. Mater..

[4] Bian J., Liu Z., Tao Y., Wang Z., Zhao X., Lin Y., Xu H., Liu Y. (2024). Advances in memristor based artificial neuron fabrication-materials, models, and applications. Int. J. Extrem. Manuf..

[5] Jiang J., Shan X., Xu J., Sun Y., Xiang T.F., Li A., Sasaki S., Tamiaki H., Wang Z., Wang X.F. (2024). Retina-Like Chlorophyll Heterojunction-Based Optoelectronic Memristor with All-Optically Modulated Synaptic Plasticity Enabling Neuromorphic Edge Detection. Adv. Funct. Mater..

[6] Chai Y. (2020). In-sensor computing for machine vision. Nature.

[7] Wang T.Y., Meng J.L., Li Q.X., He Z.Y., Zhu H., Ji L., Sun Q.-Q., Chen L., Zhang D.W. (2021). Reconfigurable optoelectronic memristor for in-sensor computing applications. Nano Energy.

[8] Zhou F., Chai Y. (2020). Near-sensor and in-sensor computing. Nat. Electron..

[9] Sun L., Qu S., Du Y., Yang L., Li Y., Wang Z., Xu W. (2022). Bio-inspired vision and neuromorphic image processing using printable metal oxide photonic synapses. ACS Photonics.

[10] Shooshtari M., Través M.J., Pahlavan S., Serrano-Gotarredona T., Linares-Barranco B. (2024). Applying Hodgkin-huxley neuron model for perovskite memristor in circuit simulation. Proceedings of the 2024 IEEE International Conference on Metrology for Extended Reality, Artificial Intelligence and Neural Engineering.

[11] Wan T., Shao B., Ma S., Zhou Y., Li Q., Chai Y. (2023). In-sensor computing: Materials, devices, and integration technologies. Adv. Mater..

[12] Ren Q., Zhu C., Ma S., Wang Z., Yan J., Wan T., Yan W., Chai Y. (2024). Optoelectronic Devices for In-Sensor Computing. Adv. Mater..

[13] Yang Q., Luo Z.D., Zhang D., Zhang M., Gan X., Seidel J., Liu Y., Hao Y., Han G. (2022). Controlled Optoelectronic Response in van der Waals Heterostructures for In-Sensor Computing. Adv. Funct. Mater..

[14] Kohn A. (2007). Visual adaptation: Physiology, mechanisms, and functional benefits. J. Neurophysiol..

[15] Webster A.M. (2015). Visual adaptation. Annu. Rev. Vis. Sci..

[16] Laughlin S.B. (1989). The role of sensory adaptation in the retina. J. Exp. Biol..

[17] Rasengane T.A., Palmer J., Teller D.Y. (2001). Infant light adaptation shows Weber’s law at photopic illuminances. Vision Res..

[18] Hecht S. (1924). The visual discrimination of intensity and the Weber-Fechner law. J. Gen. Physiol..

[19] Sakmann B., Creutzfeldt O.D. (1969). Scotopic and mesopic light adaptation in the cat’s retina. Pflugers. Arch..

[20] Liu S.C. (1997). Silicon retina with adaptive filtering properties. NeurIPS.

[21] Mahowald M.A. (1991). Silicon retina with adaptive photoreceptors/Visual information processing: From neurons to chips. SPIE.

[22] Liu W., Yang X., Wang Z., Li Y., Li J., Feng Q., Xie X., Xin W., Xu H., Liu Y. (2023). Self-powered and broadband opto-sensor with bionic visual adaptation function based on multilayer γ-InSe flakes. Light Sci. Appl..

[23] He Z., Shen H., Ye D., Xiang L., Zhao W., Ding J., Zhang F., Di C., Zhu D. (2021). An organic transistor with light intensity-dependent active photoadaptation. Nat. Electron..

[24] Xie D., Wei L., Xie M., Jiang L., Yang J., He J., Jiang J. (2021). Photoelectric visual adaptation based on 0D-CsPbBr3-quantum-dots/2D-MoS2 mixed-dimensional heterojunction transistor. Adv. Funct. Mater..

[25] He Z., Ye D., Liu L., Di C.A., Zhu D. (2022). Advances in materials and devices for mimicking sensory adaptation. Mater. Horiz..

[26] Liao F., Zhou Z., Kim B.J., Chen J., Wang J., Wan T., Zhou Y., Hoang A.T., Wang C., Kang J. (2022). Bioinspired in-sensor visual adaptation for accurate perception. Nat. Electron..

[27] Shruthi J., Jayababu N., Reddy M.V.R. (2019). Synthesis of Y_2_O_3_-ZnO nanocomposites for the enhancement of room temperature 2-methoxyethanol gas sensing performance. J. Alloy. Compd..

[28] Ansari A.A., Khan A., Labis J.P., Alam M., Aslam Manthrammel M., Ahamed M., Akhtar M.J., Aldalbahi A., Ghaithan H. (2019). Mesoporous multi-silica layer-coated Y_2_O_3_: Eu core-shell nanoparticles: Synthesis, luminescent properties and cytotoxicity evaluation. Mater. Sci. Eng. C..

[29] Porosnicu I., Butnaru C.M., Tiseanu I., Stancu E., Munteanu C.V.A., Bita B.I., Duliu O.G., Sima F. (2021). Y_2_O_3_ nanoparticles and X-ray radiation-induced effects in melanoma cells. Molecules.

[30] Louis A.J., Abdulridha A.R. (2023). Preparation of (PVA/Y_2_O_3_) Nanocomposite and Study the Optical Properties for Optoelectronic Device. J. Surv. Fish. Sci..

[31] Borrás M.C., Sluyter R., Barker P.J., Konstantinov K., Bakand S. (2020). Y_2_O_3_ decorated TiO_2_ nanoparticles: Enhanced UV attenuation and suppressed photocatalytic activity with promise for cosmetic and sunscreen applications. J. Photochem. Photobiol. B Biol..

[32] Kim H.J., Kim D.W., Lee W.Y., Kim K., Lee S.H., Bae J.H., Kang I.M., Kim K., Jang J. (2022). Flexible Sol-Gel—Processed Y_2_O_3_ RRAM Devices Obtained via UV/Ozone-Assisted Photochemical Annealing Process. Materials.

[33] Ernst W., Kemp C.M. (1975). Scotopic and photopic dark adaptation of the b wave in isolated rat retina. Nature.

[34] Jacobs G.H., Fisher S.K., Anderson D.H., Silverman M.S. (1976). Scotopic and photopic vision in the California ground squirrel: Physiological and anatomical evidence. J. Comp. Neurol..

[35] Shi L., Shi K., Zhang Z.C., Li Y., Wang F.D., Si S.H., Liu Z.B., Lu T.B., Chen X.D., Zhang J. (2024). Flexible retinomorphic vision sensors with scotopic and photopic adaptation for a fully flexible neuromorphic machine vision system. SmartMat.

[36] Shen H., He Z., Jin W., Xiang L., Zhao W., Di C.A., Zhu D. (2019). Mimicking Sensory Adaptation with Dielectric Engineered Organic Transistors. Adv. Mater..

[37] Shi J., Lin Y., Wang Z., Shan X., Tao Y., Zhao X., Xu H., Liu Y. (2024). Adaptive processing enabled by sodium alginate based complementary memristor for neuromorphic sensory system. Adv. Mater..

[38] Fain G.L., Matthews H.R., Cornwall M.C., Koutalos Y. (2001). Adaptation in vertebrate photoreceptors. Physiol. Rev..

[39] Matthews H.R. (1991). Incorporation of chelator into guinea-pig rods shows that calcium mediates mammalian photoreceptor light adaptation. J. Physiol..

[40] Chen J.Y., Huang C.W., Chiu C.H., Huang Y.T., Wu W.W. (2015). Switching kinetic of VCM-based memristor: Evolution and positioning of nanofilament. Adv. Mater..

[41] Zhang K., Li T., Liu X., Huang Z., Liu Y. (2024). The formation process and influencing factors of electric field-induced oxygen vacancy in Y_2_O_3_ transparent ceramic. Ceram. Int..

[42] Zhang R., Lin Z., Chen N., Zhao D., Chen Q. (2024). Oxygen vacancy–enriched Y_2_O_3_ nanoparticles having reactive facets for selective sensing of methyl ethyl ketone peroxide explosive. Sens. Actuator B Chem..

[43] Lee T., Kim H.I., Cho Y., Lee S., Lee W.Y., Bae J.H., Kang I.M., Kim K., Lee S.H., Jang J. (2023). Sol–Gel-Processed Y_2_O_3_ Multilevel Resistive Random-Access Memory Cells for Neural Networks. Nanomaterials.

[44] Geng X., Hu L., Zhuge F., Wei X. (2022). Retina-Inspired Two-Terminal Optoelectronic Neuromorphic Devices with Light-Tunable Short-Term Plasticity for Self-Adjusting Sensing. Adv. Intell. Syst..

[45] Wang J., Pan R., Cao H., Wang Y., Liang L., Zhang H., Gao J., Zhuge F. (2016). Anomalous rectification in a purely electronic memristor. Appl. Phys. Lett..

[46] Pan R., Li J., Zhuge F., Zhu L., Liang L., Zhang H., Gao J., Cao H., Fu B., Li K. (2016). Synaptic devices based on purely electronic memristors. Appl. Phys. Lett..

