# Supplementary material for: Photopic Adaptation Mimicked by Y2O3-Based Optoelectronic Memristor for Neuromorphic Visual System
Source: Nanomaterials (Basel). 2025 Apr 11;15(8):579. doi: 10.3390/nano15080579 (PMC12029702; doi:10.3390/nano15080579)
Supplement: Supplementary file 1 [file nanomaterials-15-00579-s001.zip › nanomaterials-3527858-supplementary.pdf]

Photopic Adaptation Mimicked by Y_2O_3-Based Optoelectronic Memristor for Neuromorphic Visual System

Jiajuan Shi †, Shanshan Qiao †, Xuanyu Shan, Zhuangzhuang Li, Zhipeng Li, Chunliang Wang *, Ye Tao,
Xiaoning Zhao, Ya Lin * and Zhongqiang Wang

Key Laboratory for UV Light-Emitting Materials and Technology, Northeast Normal University,
Ministry of Education, 5268 Renmin Street, Changchun 130024, China;
shijj969@nenu.edu.cn (J.S.); ssqiao@nenu.edu.cn (S.Q.); danxy453@nenu.edu.cn (X.S.); lizz834@nenu.edu.cn (Z.L.);
lizp394@nenu.edu.cn (Z.L.); taoy506@nenu.edu.cn (Y.T.);
zhaoxn430@nenu.edu.cn (X.Z.); wangzq752@nenu.edu.cn (Z.W.)
* Correspondence: wangcl493@nenu.edu.cn (C.W.); liny474@nenu.edu.cn (Y.L.)
† These authors contributed equally to this work.

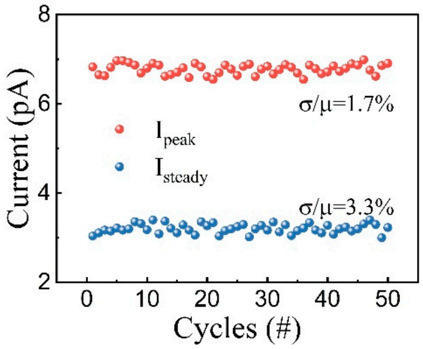

Figure S1. The statistical distribution of the I_{peak} and I_{steady} variability for 50 continuous cycles.

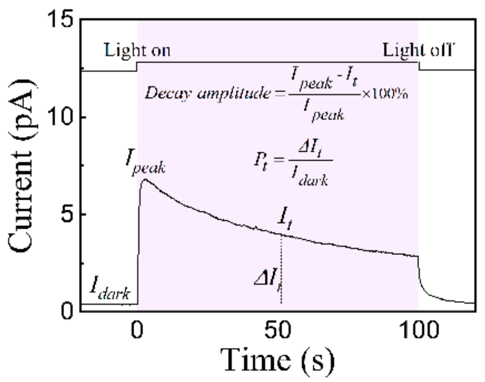

Figure S2. Definition of time-dependent photosensitivity (P_t) and Decay amplitude, where I_{dark} is the dark current, I_t is the current after t seconds of light irradiation and I_{peak} represents the under light stimulus.

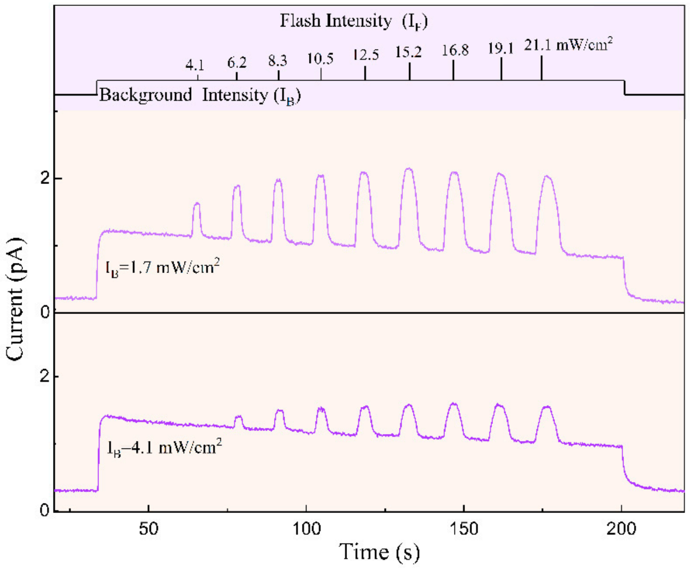

Figure S3. Current response of the Y_2O_3-based optoelectronic memristor of background stimulation (intensity of 1.7 and 4.1 mW/cm^2) and flash stimulations (intensity from 4.1 to 21.1 mW/cm^2).

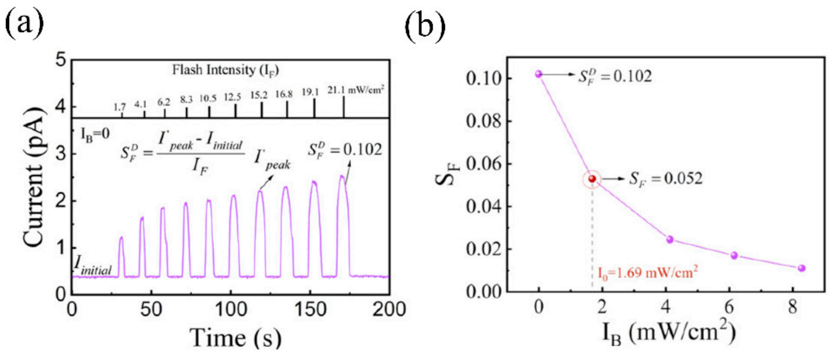

Figure S4. (a) The current response of Y_2O_3 based optoelectronic memristor under the background stimulation of 0 mW/cm^2; (b) Correlation between the sensitivity and background intensity.

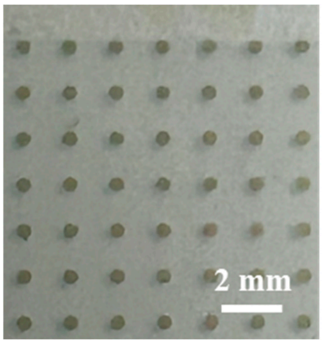

Figure S5. Photograph of a 7 × 7 optoelectronic memristive array with each pixel has an area of ~0.78 mm^2.

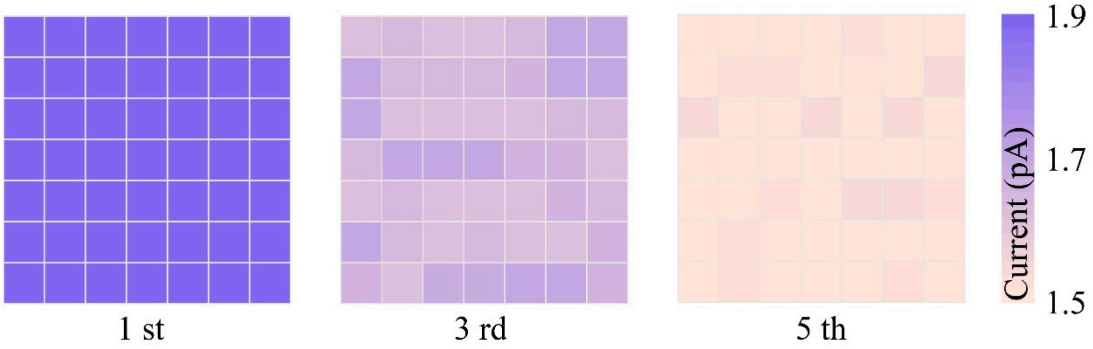

Figure S6. The process of recognizing the pattern of 'heart shape' under bright background (8.3 mW/cm^2) with flash intensity of 6.2 mW/cm^2 in 7×7 device array. With continuous light background, the 'heart shape' pattern cannot be identified from the bright background conditions.
